# Metabolic syndrome is not associated with reduction in aortic distensibility in subjects with type 2 diabetes mellitus

**DOI:** 10.1186/1475-2840-7-1

**Published:** 2008-01-02

**Authors:** Nicholas Tentolouris, Athanasia Papazafiropoulou, Ioannis Moyssakis, Stavros Liatis, Despoina Perrea, Maria Kostakis, Nicholas Katsilambros

**Affiliations:** 1 First Department of Propaedeutic Medicine, Laiko General Hospital, Athens University Medical School, 17 Agiou Thoma Street, 115 27, Athens, Greece; 2 Cardiology Department, Laiko General Hospital, 17 Agiou Thoma Street, 115 27, Athens, Greece Athens, Greece; 3 Laboratory for Experimental Surgery and Surgical Research, Athens University Medical School, 17 Agiou Thoma Street, 115 27, Athens, Greece

## Abstract

**Background:**

Aortic distensibility (AD) is a marker of the elastic properties of the aorta. Reduction of AD occurs early in subjects with type 2 diabetes mellitus (T2DM) and it is associated with subclinical generalized atherosclerosis. Metabolic syndrome (MetS) is common in subjects with T2DM and predicts cardiovascular morbidity and mortality. This study examined the potential relationship between MetS and AD in a cohort of subjects with T2DM.

**Methods and results:**

A total of 210 subjects with T2DM were studied. MetS was diagnosed using the NCEP/ATP-III criteria. AD was assessed non-invasively by ultrasonography. The prevalence of MetS was 64.8%. AD was not significantly different between subjects with and without MetS (1.80 ± 0.54 vs. 1.84 ± 0.53 10^-6 ^dyn^-1 ^cm^2^, p = 0.55). Univariate linear regression analysis showed that AD was associated positively with male sex (p = 0.02) as well as glomerular filtration rate (p < 0.001), and negatively with age (p = 0.04), history of hypertension (p = 0.001), as well as duration of diabetes (p < 0.001). After multivariate adjustment, AD was associated independently and significantly only with age (p = 0.02), duration of diabetes p < 0.001), and history of hypertension (p = 0.004); no significant relationship was found with MetS status, the sum of the components of the MetS or the individual components-besides hypertension-of the MetS.

**Conclusion:**

In subjects with T2DM, MetS status *per se *is not associated with reduction of AD. In addition, it was shown that besides ageing, duration of glycemia was a strong predictor of AD. From the components of the MetS only hypertension was associated with reduction of the elastic properties of the aorta.

## Background

Patients with type 2 diabetes mellitus (T2DM) suffer often from macrovascular complications [1] while cardiovascular morbidity and mortality is particularly common in this group of patients [2]. Previous studies have shown that aortic distensibility (AD), an index of aortic elasticity, is lower in individuals with T2DM in comparison with age- and sex-matched nondiabetic counterparts [3]. AD can be determined with high accuracy non-invasively using ultrasonography [4] and is considered an index of the total arterial stiffness and an important cardiovascular risk factor [5].

Metabolic syndrome (MetS) is defined as the clustering of cardiovascular risk factors such as increased blood pressure, insulin resistance, dyslipidaemia, central obesity, high triglyceride and low high density lipoprotein (HDL) cholesterol concentrations [6]. Presence of MetS is associated with increased risk for cardiovascular morbidity and mortality [7] as well as development of T2DM [8]. Two recently published studies have shown that in the general population MetS is associated with increased carotid intima-media thickness (IMT) [9] and pulse wave velocity (PWV) [10], which reflect structural and functional properties of the arteries, respectively. However, the effect of MetS on the elastic properties of the arteries in subjects with T2DM is not well known. This cross-sectional study examined the potential association between AD and MetS in a cohort of T2DM subjects.

## Methods

### Subjects

A total of 210 subjects with T2DM, consecutively selected from the diabetes outpatient clinic of our hospital were enrolled into the study. Diagnosis of diabetes was based on the American Diabetes Association criteria [11]. A detailed history for the presence of cardiovascular disease, current medication, information about other diseases and smoking habits was obtained, and a thorough physical examination was performed.

All measurements were performed in the morning, after 10–12 hours fast. The subjects were advised not to eat, smoke, or drink coffee before examination. Blood samples were drawn for measurement of plasma glucose, glycosylated haemoglobin A1c (HbA_1c_), creatinine, and lipid profile. The antidiabetic medications were given to the patients at the end of the examination. The purpose of the study was clearly explained to all subjects, who then volunteered to participate. The ethics committee of our hospital approved the study and informed written consent was obtained.

Blood pressure was measured three consecutive times, one minute in apart, in the sitting position using an appropriate cuff size. The mean values of the last two measurements was calculated and used in the analysis. Arterial hypertension was defined according to the current guidelines [12], when systolic was ≥ 140 mm Hg or and/or diastolic blood pressure was ≥ 90 mm Hg or when the patients were on antihypertensive treatment. In addition the type of the antihypertensive treatment was recorded. Of the study subjects 21.2% was on treatment with angiotensin-converting enzyme inhibitors (ACE-I) and/or angiotensin II receptor blockers (ARBs), 4.3% on diuretics, 7.1% on β-blockers, and 9% on calcium channel blockers. Coronary artery disease was defined as presence of angina, history of previous myocardial infarction, positive stress testing, revascularization procedures or stenosis > 50% at the coronary arteries. Direct fundoscopy was performed in all patients through dilated pupils.

Body weight with subjects in light clothing without shoes and height was measured and body mass index (BMI) was calculated. Waist circumference was measured with a soft tape on standing, midway between the lowest rib and the iliac crest. Hip circumference was measured over the widest part of the gluteal region, and then the waist-to-hip ratio (WHR) was calculated.

### Definition of the MetS

Subjects having three or more of the following criteria, according to the National Cholesterol Education Program/Adult Treatment Panel III (NCEP/ATP III) report [12], were defined as having the MetS: abdominal obesity (waist circumference > 102 cm in men and > 88 cm in women), triglycerides ≥ 1.7 mmol/L, low HDL cholesterol: ≤ 1.0 mmol/L in men and ≤ 1.3 mmol/L in women, high blood pressure: ≥ 130/85 mm Hg or use of antihypertensive drugs and high fasting plasma glucose: ≥ 6.1 mmol/L or diagnosed diabetes mellitus.

### Analytical methods

Fasting serum glucose, lipids and creatinine concentrations were measured on a Technicon analyzer RA-XT. Low density lipoprotein (LDL) cholesterol concentrations were calculated using the Friedewald formula [13]. HbA_1c _was measured by high-performance liquid chromatography (HPLC) (Roche Diagnostics, Mannheim, Germany) with a non-diabetic reference range of 4.0–6.0%. Microalbuminuria was assessed by measuring the albumin-to-creatinine ratio (ACR) in a random urine sample on a DCA 2000 analyzer using the immunonephelometry technique (Bayer HealthCare LLC, Elkhart, USA). Glomerular filtration rate (GFR) was calculated using the Modification of Diet in Renal Disease formula [14]. Plasma insulin was measured with radioimmunoassay (Biosure, Belgium; c.v. = 3.3 ± 1.2%). Insulin resistance was estimated using the homeostasis model assessment equation of insulin resistance (HOMA-IR) [15].

### Determination of AD

AD was determined non-invasively based on the relationship between changes in aortic diameter and pressure with each cardiac pulse [4]. The echocardiographic study was carried out using a Hewlett Packard Sonos 1000 ultrasound system (Hewlett Packard, USA), using a 2.5 MHz transducer. Each subject was placed in the mild left recumbent position and the ascending aorta was recorded at a level 3 cm above the aortic valve in the M-mode tracings guided by the two-dimensional echocardiogram in the parasternal long axis view [4]. Internal aortic diameters were measured by means of a caliper in systole and diastole as the distance between the trailing edge of the anterior aortic wall and the leading edge of the posterior aortic wall. Systolic aortic diameter was measured as the maximal anterior motion of the aorta and diastolic diameter at the peak of the QRS complex on the simultaneously recorded electrocardiogram. Blood pressure was measured three consecutive times, one minute in apart at the heart level during echocardiographic examination; the mean values of the last two measurements was calculated and used in the analysis. In addition, aortic diameter was measured for ten consecutive cardiac beats routinely and averaged. AD was calculated according to the formula [4,16]:

Aortic distensibility=2ΔDDd⋅(Ps−Pd)10−6 dyn−1 cm2
 MathType@MTEF@5@5@+=feaagaart1ev2aaatCvAUfKttLearuWrP9MDH5MBPbIqV92AaeXatLxBI9gBaebbnrfifHhDYfgasaacPC6xNi=xH8viVGI8Gi=hEeeu0xXdbba9frFj0xb9qqpG0dXdb9aspeI8k8fiI+fsY=rqGqVepae9pg0db9vqaiVgFr0xfr=xfr=xc9adbaqaaeGacaGaaiaabeqaaeqabiWaaaGcbaGaeeyqaeKaee4Ba8MaeeOCaiNaeeiDaqNaeeyAaKMaee4yamMaeeiiaaIaeeizaqMaeeyAaKMaee4CamNaeeiDaqNaeeyzauMaeeOBa4Maee4CamNaeeyAaKMaeeOyaiMaeeyAaKMaeeiBaWMaeeyAaKMaeeiDaqNaeeyEaKNaeyypa0tcfa4aaSaaaeaacqaIYaGmcqqHuoarcqWGebaraeaacqWGebarcqWGKbazcqGHflY1cqGGOaakcqWGqbaucqWGZbWCcqGHsislcqWGqbaucqWGKbazcqGGPaqkaaGccqaIXaqmcqaIWaamdaahaaWcbeqaaiabgkHiTiabiAda2aaakiabbccaGiabbsgaKjabbMha5jabb6gaUnaaCaaaleqabaGaeyOeI0IaeGymaedaaOGaeeiiaaIaee4yamMaeeyBa02aaWbaaSqabeaacqaIYaGmaaaaaa@68D4@, where ΔD is the change of the aortic diameter between systole and diastole, Dd is the aortic diameter in diastole, Ps is the systolic and Pd is the diastolic blood pressure.

All measurements were performed by the same experienced cardiologist, who was blind for the status of the participants. The intraobserver coefficient of variation was examined in our laboratory in 20 randomly selected healthy subjects and was 4.2% for the systolic and 4.1% for the diastolic aortic dimensions, respectively (not significant differences).

The intra-observer mean percentage error (absolute difference between two observations divided by the mean and expressed as percentage) was determined for the AD in 20 randomly selected subjects and was 7.5% in our center.

### Statistical analysis

Statistical analysis was preformed using programs available in the SPSS statistical package (SPSS 12.0, Chicago, USA). All variables were tested for normal distribution of the data. Data are shown as mean ± SD, unless it is stated otherwise. A two sample *t*-test was used to assess differences in continuous variables, while a chi-square test was used for categorical variables. Univariate linear regression analysis was performed to look for the relationship between AD and the variables of interest in the sample population. Then, multivariate linear regression analyses were performed (backward stepwise method) to look for independent associations between AD and the variables of interest. A total of 7 models of multivariate linear regression analysis have been created. Model 1 (core model) included the variables that were found to be associated significantly with AD in univariate analysis and included age, sex, duration of diabetes, history of hypertension, and GFR; models 2–7 included the variables of the core model and, in addition, MetS status (model 2), the sum of the components of the MetS (model 3), high waist circumference (model 4), low HDL cholesterol (model 5), high triglycerides (model 6), and high blood pressure (model 7). All independent variables in the multivariate analyses models were tested for multicolinearity. *P *< 0.05 (two-tailed) was considered statistically significant.

## Results

Prevalence rate of MetS was 64.8% in the study group. Patients with and without MetS did not differ in terms of age and sex. Diabetic patients with MetS had higher values of waist, WHR, blood pressure, triglycerides, GFR, and lower HDL cholesterol values in comparison with the diabetic subjects without the MetS. In addition, those with MetS had more often hypertension and microalbuminuria (Table 1). Moreover, diabetic patients with MetS were more insulin resistant, although the difference was not statistically significant (Table 1).

**Table 1:** Demographic and clinical characteristics of the study subjects stratified according to the presence (MetS+) or not (MetS-) of metabolic syndrome

	MetS (-)	MetS (+)	p
n (%)	74 (35.2)	136 (64.8)	-
Males/females n (%)	35 (52.7)/39 (47.3)	72 (52.9)/64 (47.1)	0.98
Age (years)	59.9 ± 7.4	60.8 ± 8.4	0.43
Body mass index (kg/m^2^)	27.6 ± 4.8	28.8 ± 5.78	0.12
Waist (cm)	94.8 ± 11.1	103.9 ± 11.5	<0.001
Waist-to-hip ratio	0.91 ± 0.08	0.95 ± 0.069	<0.001
Systolic blood pressure (mm Hg)	128.2 ± 22.7	142.9 ± 18.4	<0.001
Diastolic blood pressure (mm Hg)	74.38 ± 12.66	81.05 ± 11.32	<0.001
Pulse pressure (mm Hg)	53.8 ± 17.0	61.2 ± 15.8	0.008
Glucose (mmol/l)	9.37 ± 3.46	9.12 ± 2.68	0.63
Total cholesterol (mmol/l)	5.55 ± 1.14	5.67 ± 1.29	0.52
HDL cholesterol (mmol/l)	1.33 ± 0.33	1.03 ± 0.22	<0.001
LDL cholesterol (mmol/l)	3.66 ± 1.03	3.71 ± 1.09	0.91
Triglycerides (mmol/l)	1.14 ± 0.42	1.98 ± 1.04	<0.001
GFR (ml/min/1.73 m^2^)	99.7 ± 26.8	107.5 ± 38.9	0.35
HOMA-IR*	3.7 (2.5–6.1)	4.9 (3.2–8.3)	0.10
Duration of diabetes (years)	10.28 ± 5.93	11.53 ± 8.78	0.31
HbA1c (%)	7.78 ± 1.68	7.55 ± 1.74	0.35
Hypertension (yes) n (%)	25 (47.2)	69 (68.3)	0.03
CAD (yes) n (%)	4 (8)	12 (12.9)	0.24
Use of stains (yes) n (%)	9 (12.2)	35 (25.7)	0.02
Any retinopathy (yes) n (%)	5 (6.8)	19 (14.5)	0.08
Microalbuminuria (yes) n (%)	16 (32)	47 (51.1)	0.01
Treatment for diabetes n (%)			0.49
Diet alone	8 (10.8)	22 (16.4)	
Antidiabetic tablets	54 (73)	88 (65.7)	
Insulin	12 (16.2)	24 (17.9)	

*Median values (interquartile range). *p *values for the comparison between groups with and without metabolic syndrome by independent samples t-test for continuous variables, Mann-Whitney U test for ordinal data or by Pearson χ^2 ^for nominal variables.

HDL: high density lipoprotein; LDL: low density lipoprotein; HOMA-IR: homeostasis model assessment insulin resistance; GFR: glomerular filtration rate; CAD: coronary artery disease.

AD values was not different between diabetic patients with and without the MetS (1.80 ± 0.54 vs. 1.84 ± 0.53 10^-6 ^dyn^-1 ^cm^2^, p = 0.55). Moreover, women with diabetes had significantly lower values of AD than diabetic men (1.74 ± 0.36 vs. 1.86 ± 0.41 10^-6 ^dyn^-1 ^cm^2^, p = 0.02). Furthermore, patients with, in comparison with those without history of hypertension, had lower AD (1.76 ± 0.18 vs. 1.87 ± 0.20 10^-6 ^dyn^-1 ^cm^2^, respectively, p = 0.001).

Univariate linear regression analysis showed that AD was associated significantly with sex [male vs. female, standardized regression coefficient (*beta*) = 0.15, p = 0.02), age (*beta *= -0.14, p = 0.04), history of hypertension (*beta *= -0.27, p = 0.001), duration of diabetes (*beta *= -0.49, p < 0.001), and GFR (*beta *= 0.33, p < 0.001). Concerning the relationship between AD and the MetS status or the particular components of the MetS, the same analysis showed no significant association between AD and MetS status (p = 0.55) or the following components of the MetS: high waist circumference [n = 124 (59.0%), p = 0.21), low HDL cholesterol levels [n = 127 (60.5%), p = 0.33], and high triglycerides levels [n = 82 (39.0)%, p = 0.55]; however, there was a suggestive association with high blood pressure [n = 165 (78.6%), *beta *= -0.13, p = 0.07] and with the sum of the components of the MetS (*beta *= -0.14, p = 0.054). In addition, no any significant association was found with smoking status (p = 0.48), BMI (p = 0.96), HbA_1c _(p = 0.44), presence of retinopathy (p = 0.26), ACR (p = 0.43), HOMA-IR (p = 0.98), use of statins (p = 0.89), and use of various classes of antihypertensive medications like ACE-I/ARBs, β-adrenergic blockers, calcium channel blockers or diuretics (all p > 0.05).

Multivariate linear regression analysis demonstrated, after controlling for GFR, significant independent associations between AD and age (p = 0.02), duration of diabetes (p < 0.001), and history of hypertension (p = 0.004) and a suggestive association with sex (p = 0.08). These variables explained 34% of the variance of AD. No any significant relationship was found with MetS status, sum of the components of the MetS, or with the individual components-besides hypertension-of the MetS (Table 2).

**Table 2:** Multivariate linear regression analysis: the association between various parameters with aortic distensibility in the study population.

	B	SE (B)	beta	p	Adjusted R^2^
**Independent variables**					
**Model 1**					
Age (1 year)	-0.004	0.002	-0.17	0.02	
Sex (male vs. female)	0.046	0.026	0.12	0.08	
Duration of diabetes (1 year)	-0.009	0.002	-0.36	<0.001	
History of hypertension (yes vs. no)	-0.080	0.027	-0.20	0.004	0.34
**Model 2**					
MetS status (yes vs. no)	-0.010	0.036	-0.019	0.78	0.33
**Model 3**					
Sum of the components of the MetS	-0.015	0.013	-0.084	0.24	0.34
**Model 4**					
High waist (yes vs. no)	-0.036	0.029	-0.09	0.21	0.33
**Model 5**					
Low HDL cholesterol (yes vs. no)	-0.026	0.027	-0.09	0.33	0.33
**Model 6**					
High triglycerides (yes vs. no)	-0.017	0.029	-0.04	0.55	0.33
**Model 7**					
High blood pressure (yes vs. no)	-0.065	0.036	-0.13	0.07	0.34

Parentheses show the units of increment or the categories of the independent variables.

B: unstandardized regression coefficient; SE: standard error; beta: standardized regression coefficient; MetS: metabolic syndrome; HDL: high density lipoprotein. All models were adjusted in addition for glomerular filtration rate.

## Discussion

### Prevalence of MetS in T2DM

As expected and in agreement with previous reports, the present study showed that MetS is common (64.8%) among subjects with T2DM [17]. Even higher rates (75–78%) of MetS in diabetic subjects have been described previously [18,19]. Published data on the prevalence of MetS among subjects with diabetes in Greece are limited [20].

### MetS and early atherosclerosis in diabetes

The main finding of the present study is that in patients with T2DM, AD is not different between those with and without MetS and that diabetes-related factors, like duration of the disease as well as hypertension, are strongly associated with AD. This finding suggests that, from the clinical point of view, presence of MetS *per se *does not add information in the identification of early atherosclerosis in patients with T2DM.

One recent large-scale study demonstrated that although MetS was associated with marginally higher carotid artery stiffness in patients with clinical manifestations of arterial disease, after multivariate adjustment, the relationship between carotid stiffness and MetS waned, while diabetes status emerged as independent predictor of carotid artery stiffness [21]. Another study in Indigenous Australians showed that diabetes status and some of the components of the MetS were associated with carotid artery IMT in this high-risk population for cardiovascular disease mortality [22]. In addition, recent data demonstrated that although intensive diabetes control was associated with higher prevalence of MetS in subjects with type 1 diabetes, glycemic control, but not MetS status, predicted the development of micro-and macro-vascular in this group of patients [23]. Moreover, other data showed that cardiovascular or all-cause mortality was not different in subjects with T2DM stratified according to MetS status [18], and that MetS was not associated independently with coronary artery disease, while diabetes status did [24]. Thus, our data corroborate these reports showing that among patients with T2DM, MetS *per se *is not associated with macrovascular complications.

### Diabetes-related factors are strongly associated with AD

Multivariate analysis demonstrated an independent relationship between AD and the known duration of diabetes. A large body of evidence exists suggesting that diabetes affects the functional properties of the aorta [25,26], overcoming the potential effect of other cardiovascular risk factors. Reduction of AD occurs early in patients with T2DM, and one study showed that AD is low even in the nondiabetic first degree relatives of patients with T2DM [25]. Chronic exposure of the aorta to the effects of hyperglycaemia explains the detrimental effect of diabetes on reduction of the elastic properties [25]. Non-enzymatic glycation of matrix proteins caused by chronic hyperglycaemia results in structural and functional changes of the aorta [26,27]. Moreover, *in vitro *glycation of collagen and elastin, and accumulation of advanced glycation end-products have been shown to increase arterial stiffness [26]. Furthermore, the present study has shown that diabetic women had stiffer aortas than diabetic men. This finding is in accordance with previous reports showing that diabetic women with either type 1 [28] or type 2 diabetes [29,30] have stiffer arteries in comparison with men with diabetes.

### The effect of hypertension on AD in diabetes

Besides duration of diabetes, history of hypertension was an important determinant of AD. It is known that from the traditional cardiovascular risk factors, hypertension is much more common in subjects with T2DM. The UK Prospective Diabetes Study demonstrated the importance of the strict blood pressure control in the reduction of microvascular and macrovascular complications in subjects with T2DM [31]. The Multiple Risk Factor Intervention Trial (MRFIT) showed that diabetic patients with increased blood pressure had a two-fold higher annual death rate in comparison with normotensive subjects [1]. Other studies demonstrated that blood pressure was the strongest component of the MetS associated with reduction of the arterial elasticity [10,32]. Furthermore, in agreement with previous reports we showed that ageing is strongly associated with reduction of the elastic properties of the aorta, irrespective of the presence of diabetes [33,34].

It is noteworthy that patients with T2DM and MetS had more often microalbuminuria in comparison with those without MetS. This finding is not surprising, given that microalbuminuria was included in the diagnostic criteria for the MetS proposed by the World Health Organization [35]. However, microalbuminuria was not associated with AD in subjects with diabetes in either univariate or multivariate analysis, suggesting that, contrary to the non-diabetic population [32], diabetes may overcome the effect of microalbuminuria on the elastic properties of the arteries. Interestingly, despite the recommendations for low LDL cholesterol and blood pressure levels in the patients with diabetes, only a small percentage of them have been treated with lipid lowering medications and only 12.8% of those had LDL cholesterol levels < 2.6 mmol/l and 47.7% had systolic/diastolic blood pressure levels at the recommended target < 130/80 mm Hg.

## Limitations

Our study has some limitations. First, many diabetic patients were on treatment with statins. Therefore, prevalence rate of dyslipidaemia and MetS may have been underestimated in the diabetic group, although statins lower mainly the LDL-cholesterol, which is not a component of the MetS. Second, in this study aortic diameters were measured echocardiographically. Previous studies have shown that aortic diameter can be obtained with a high degree of accuracy in subjects whose cardiothoracic anatomy permits an echocardiographic signal of satisfactory quality [4]. Moreover, the pulse pressure obtained by external sphygmomanometer introduces an error factor, but the final result in the validity of the aortic distensibility calculation is trivial [4].

## Conclusion

In conclusion, our study showed that in subjects with T2DM, MetS status *per se *is not associated with reduction in AD. In addition, we showed that besides ageing, duration of glycemia was strong predictor of AD. From the components of the MetS only hypertension was associated with reduction of the elastic properties of the aorta.

## Abbreviations

AD: Aortic distensibility;

MetS: Metabolic syndrome;

T2DM: Type 2 diabetes mellitus;

IMT: Intima-media thickness;

PWV: Pulse wave velocity;

HDL: High density lipoprotein;

ACE-I: Angiotensin-converting enzyme inhibitors;

ARBs: Angiotensin II receptor blockers;

BMI: Body mass index;

WHR: Waist-to-hip ratio;

HbA_1c_: Glycosylated haemoglobin A1c;

NCEP/ATP III: National Cholesterol Education Program/Adult Treatment Panel III;

LDL: Low density lipoprotein;

HPLC: High-performance liquid chromatography;

ACR: Albumin-to-creatinine ratio;

GFR: Glomerular filtration rate;

HOMA-IR: Homeostasis model assessment equation of insulin resistance;

ΔD: The change of the aortic diameter between systole and diastole;

Dd: Aortic diameter in diastole;

Ps: Systolic blood pressure;

Pd: Diastolic blood pressure.

## Competing interests

The author(s) declare that they have no competing interests.

## Authors' contributions

NT: wrote the protocol and manuscript; contacted participants, performed medical examination in part of the participants, performed statistical analysis, corrected the final version of the manuscript

AP: wrote the draft manuscript, performed medical examination in part of the participants, provided informed consent to the study subjects, participated in the collection and storage of blood samples, corrected the final version of the manuscript

IM: performed all ultrasound examinations and calculated aortic distensibility

SL: contact with participants, performed medical examination in part of the participants, provided informed consent to the study subjects, participated in blood sample collections and storage, corrected the final version of the manuscript

DP: performed all analytical methods, wrote the analytical analysis section, corrected the final version of the manuscript

MK: performed medical examination in part of the participants, participated in blood sample collections and storage, corrected the final version of the manuscript

NK: Participation in the design of the study, corrected the protocol and the final version of the manuscript.
